# Broadband circular dichroism in chiral plasmonic woodpiles

**DOI:** 10.1007/s00339-023-06481-9

**Published:** 2023-03-02

**Authors:** Bilel Abdennadher, René Iseli, Ullrich Steiner, Matthias Saba

**Affiliations:** Adolphe Merkle Institute, University of Fribourg, Chemin des Verdiers 4, Fribourg, 1700 Switzerland

**Keywords:** Complex bandstructure, Circular dichroism, Plasmonic crystal, Chiral woodpile, Thermal emission

## Abstract

The circular dichroism (CD) of a material is the difference in optical absorption under left- and right-circularly polarized illumination. It is crucial for a number of applications, from molecular sensing to the design of circularly polarized thermal light sources. The CD in natural materials is typically weak, leading to the exploitation of artificial chiral materials. Layered chiral woodpile structures are well known to boost chiro-optical effects when realized as a photonic crystal or an optical metamaterial. We here demonstrate that light scattering at a chiral plasmonic woodpile, which is structured on the order of the wavelength of the light, can be well understood by considering the fundamental evanescent Floquet states within the structure. In particular, we report a broadband circular polarization bandgap in the complex band structure of various plasmonic woodpiles that spans the optical transparency window of the atmosphere between 3 and 4 $\upmu$m and leads to an average CD of up to 90% within this spectral range. Our findings could pave the way for an ultra-broadband circularly polarized thermal source.

## Introduction

The manipulation of light through optical elements such as lenses, color filters, and polarizers goes back to ancient times and can ubiquitously be found in the living world [1, 2]. The limits that naturally available materials impose on light manipulation can be overcome by nano-structuring matter in form of for example photonic crystals, dielectric geometries that are structured on the order of the wavelength of the light and lead to interference effects [3, 4]. A different strategy to obtain new optical materials employs plasmonic metals structured on a deeply sub-wavelength scale, so-called metamaterials [5]. A vast number of chiral photonic crystal and metamaterial designs [6] have been suggested to yield custom-designed chiro-optical effects, such as strong circular dichroism [7–12], optical activity [13–18], and orbital angular momentum generation [19–21]. Next to a number of applications in sensing, catalysis, and chiral light generation, when realized on a micrometer length scale, these chiral geometries can be engineered to yield circularly polarized thermal emission by application of Kirchhoff’s law [22–32].

**Fig. 1:**
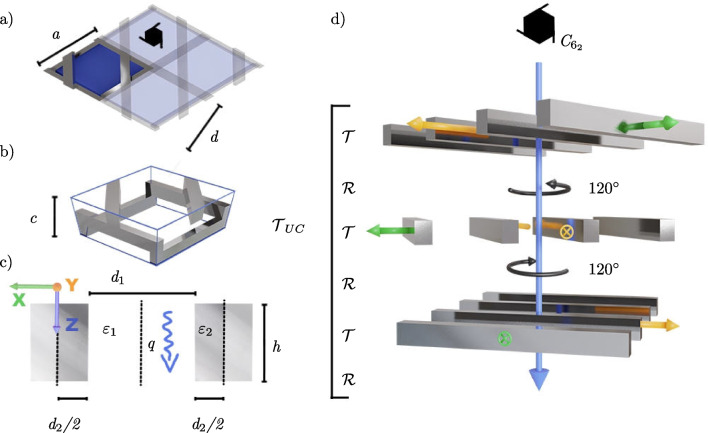
The hexagonal chiral woodpile plasmonic crystal. **a** Bird’s eye view onto the Wigner–Seitz cell with hexagonal lattice constant *a* and single layer pitch *d*. **b** Side view showing the vertical pitch *c*. **c** Each plasmonic bar with permittivity $\varepsilon _2$ has a width $d_2\,{=}\,\phi \,d$ and a height $h\,{=}\,c/3$. The dashed lines show the layer mirror planes in the center of the dielectric and the metal region. **d** The global $C_{6_2}$ symmetry of the underlying $P6_222$ (180) space group [33] is generated through an active $C_3$ (120$^\circ$) rotation of the coordinate frame ${\mathcal {R}}$ after each lamellar layer. The unit cell transfer matrix is thus given by ${\mathcal {T}}_\mathrm{{UC}}\,{=}\,({\mathcal {R}}{\mathcal {T}})^3$, with the transfer matrix through one lamellar grating layer ${\mathcal {T}}$

A particularly promising and relatively easy to manufacture (down to sub-micrometer length scales) geometry is the chiral woodpile [34–38] as illustrated in Fig. 1. When realized as a classical deeply sub-wavelength metamaterial, the chiral woodpile, which is evidently not based on local meta-atoms, does not show a strong chiro-optical response due to the mismatch between the screw-axis pitch and the vacuum wavelength. A hexagonal chiral woodpile realized as a lossy photonic crystal with big index contrast (using a semiconductor with $\varepsilon \,{=}\,8.9$), on the other hand, is predicted to produce circularly polarized thermal emission within a broad band (gap-to-midgap ratio $\Delta \omega /\langle \omega \rangle \approx 1/6$) [22]. While a more sophisticated fully three-dimensional geometry with $\varepsilon \,{=}\,12$ is predicted to improve the bandwidth to a gap-to-midgap ratio of 1/3 [12], we here instead consider a plasmonic hexagonal woodpile structured on the order of the wavelength that leads to a strongly circularly polarized thermal emission within a band with a gap-to-midgap ratio ${\gtrsim }\,2/7$, focusing on the optical transparency window of the atmosphere between 3 and 4$\upmu$m. A similar structure has been previously considered to engineer linearly polarized thermal emission [36]. Since the spatial periodicity of such a *plasmonic crystal* (PC) is on the order of the wavelength, standard metamaterial homogenization techniques cannot be applied. Looking at the field patterns can shed some light on the chiral plasmonic excitations, as for example done for a helix PC [39].

The simulated scattering of an outside source, however, always corresponds to an a-priory unknown superposition of self-consistent modal solutions within the PC. A physically inspired set of such modal solutions is provided through evanescent Floquet states [40–42]. These states can be thought of as a natural complex generalization of the Bloch modes discussed in the canonical band structure picture employed in the photonic crystal theory [3]. They thus allow to account for base materials with general optical permittivity and permeability and span the solution space of the monochromatic Maxwell equations within finite slab-like geometries with periodicity. While the calculation of evanescent Floquet modes is not implemented in standard simulation tools based on the finite element or finite difference methods, they can be naturally obtained through a plane-wave approach [40] for photonic crystals. For plasmonic materials, such an approach is, however, very inefficient as the generally poor convergence behavior of the plane-wave basis becomes a particularly big problem at metal-dielectric interfaces [41], where the permittivity changes its sign. In simple geometries, which are homogeneous in the propagation direction, such as the lamellar grating [43], the fishnet structure [42], or hyperbolic aligned wire media [44, 45], the computation simplifies to an essentially one- or two-dimensional problem. More generally, in a geometry that is made by a number of slices that are individually homogeneous in the propagation direction, the evanescent Floquet modes are efficiently calculated as the eigen-solutions of the corresponding transfer matrix through the unit cell [46–50].

We here show that the fundamental Floquet modes responsible for propagating energy through (and absorbing it within) a single lamellar grating layer are sufficient to calculate the two fundamental Floquet modes of a hexagonal chiral woodpile made of a sequence of equal lamellar grating layers, copied through a $C_{6_2}$ screw rotation, as illustrated in Fig. 1d. These fundamental Floquet states of the chiral woodpile in turn describe the scattering physics well and reveal the origin of a broad circularly polarized stop band in the absorption spectrum, which is induced through a circularly polarized pseudo-bandgap. We use these two Floquet modes as a scattering basis within the fundamental Bragg order to efficiently optimize the broadband circular dichroism and derive general design principles based on the thickness of the dielectric region $d_1$ and the chiral pitch *c* only (Fig. 1). We show that the obtained absorption spectra are surprisingly accurate considering the strong approximations made and agree well with full-wave simulations. Our findings suggest experimental investigation of the designed woodpiles for chiral thermal emission. More fundamentally, they demonstrate the power of the concept of evanescent Floquet states to understand light propagation in finite slabs of artificially structured materials.

## Methods

Evanescent Floquet states span the vector space of solutions of Maxwell’s equations within a finite slab of a periodic material [40]. While in theory, a countably infinite number of such modes exists at each frequency, a small number usually suffices in practice, making them an invaluable tool to not only predict but most importantly understand the physical origin of a scattering experiment. If a periodic optical material contains base materials with loss, such as plasmonic metals, even the fundamental Floquet modes do not fit into the standard band structure picture with real-valued wave vectors [3]. Instead, a complex band structure picture has to be adopted [40, 41]. Calculating these modes for general geometries remains challenging and is currently not possible with established software packages (based on for example finite differences or finite elements). We here present a semi-analytical method to efficiently compute the complex band structure for the fundamental evanescent Floquet states of a hexagonal chiral woodpile. While our method cannot accurately predict scattering observables, it instead

1. reveals the main physical mechanism of scattering even for frequencies close to the first Wood anomaly substantially above the homogenization regime, and
2. allows for easily optimizing a desired behavior (broadband circular dichroism in this manuscript) within a large parameter space.

### Single layer scattering

We start by calculating the Floquet states within each layer, which is homogeneous in *z* direction and whose local coordinate frame is defined in Fig. 1c. The procedure is based on a symmetry simplification of the well-known lamellar grating equation [43]. At normal incidence, the electric (TE) or magnetic (TM) field points in *y*-direction, and is thus anti-symmetric with respect to the mirror operation $\sigma _y$ that maps $y\,{\mapsto }\,{-}y$. The relevant scalar (monochromatic) field $F(\textbf{r})$ is the *y*-component of the electric (TE) or magnetic (TM) field. For the whole chiral slab, since we are interested in plane-wave excitation at normal incidence, we can characterize the field as odd with respect to the two-fold rotation symmetry around the *z*-axis at the center of the unit cell $C_{2z}$, that coincides with and results from multiple application of the $C_{6_2}$-axis shown in cf. Fig. 1d. The scalar field $F(\textbf{r})$ is, therefore, symmetric under $\sigma _x\,{=}\,C_{2z}\sigma _y$ at the center of both the dielectric and the metal domain, shown as dashed lines in Fig. 1c.

Due to the homogeneity of the single layer in *z*-direction, the Floquet modes are plane-wave-like. The general monochromatic solution of Maxwell’s equations that satisfies the symmetry requirements in the domain $\eta \,{=}\,1,2$, where $1\,{\equiv }\,\text {dielectric}$ and $2\,{\equiv }\,\text {metal}$, is hence (with $\imath \,{:=}\,\sqrt{{-}1}$)

1 $$\begin{aligned} F_{\eta }(\textbf{r}) = c_{\eta }\,\cos \left[ k_{\eta }(x-x_{\eta })\right] \,e^{\imath q z} . \end{aligned}$$

Here, $c_{\eta }\,{\in }\,{\mathbb {C}}$ is a complex coefficient, $q\,{\in }\,{\mathbb {C}}$ the wave number in the propagation direction, $x_{\eta }$ the center of the domain, and $k_{\eta }\,{=}\,\pm \sqrt{\varepsilon _{\eta } k_0^2-q^2}$ the lateral wave number given by the material dispersion relation, with $k_0\,{:=}\,\omega /c_0$ the vacuum wave number (with $\omega$ the angular frequency and $c_0$ the speed of light). The solution at the interface between the two domains additionally requires the tangential components of the electric and the magnetic field to be continuous, which leads to

2a $$\begin{aligned} c_1\,\cos \left[ \varphi _1\right]&= c_2\,\cos \left[ \varphi _2\right] \end{aligned}$$

2b $$\begin{aligned} Z_1c_1\,\sin \left[ \varphi _1\right]&= -Z_2c_2\,\sin \left[ \varphi _2\right] \text { ,} \end{aligned}$$

with the wave impedance $Z_{\eta }\,{=}\,k_{\eta }/k_0$ (TE) and $Z_{\eta }\,{=}\,k_{\eta }/(\varepsilon _{\eta }k_0)$ (TM) and the (generally complex-valued) optical phase $\varphi _{\eta }\,{=}\,k_{\eta }d_{\eta }/2$. A countably infinite number of Floquet solutions is thus obtained from solving the root equation

3 $$\begin{aligned} 0&= \lambda (q) \nonumber \\&:= Z_1\sin \left[ \varphi _1\right] \cos \left[ \varphi _2\right] + Z_2\sin \left[ \varphi _2\right] \cos \left[ \varphi _1\right] . \end{aligned}$$

This is evidently a transcendental problem as all $\varphi _{\eta }$ and $Z_{\eta }$ implicitly depend on *q* through the respective material dispersion relation. For the metal-dielectric structure and the wavelength range under consideration, however, a good guess can be obtained analytically. For this, let us first discuss the function $\lambda (q)$ in the complex plane. It inherits two branch points from the two root functions of $k_{\eta }$ at $q_{\eta }\,{=}\,\sqrt{\varepsilon _{\eta }} k_0$.

We now use the fact that these two roots are well separated (compared to $k_0$) in the mid-infrared, where $\Vert q_2\Vert \gg \Vert k_0\Vert$ for all metals. Due to the impedance mismatch between the two regions, the intensity of the low-order Floquet modes is either concentrated in the dielectric or in the metal region. In other words, solutions will be either found relatively close to $q_1$ or close to $q_2$ (and far away from the other branch point). Let us start with the first case, where the intensity is concentrated in the dielectric. Since the solution is far away from $q_2$, the phase $\varphi _2$ has a large, positive imaginary part $\Im [\varphi _2]\,{\gg }\,1$. The root function thus simplifies to

$$\begin{aligned} \lambda (q)\approx Z_1\sin [\varphi _1]+\imath Z_2\cos [\varphi _1]. \end{aligned}$$

For TE polarization, $\Vert Z_2\Vert \,{\gg }\,\Vert Z_1\Vert$, while for TM polarization, $\Vert Z_1\Vert \,{\gg }\,\Vert Z_2\Vert$, so that we obtain

4 $$\begin{aligned} q_1^{(\alpha )}&= \sqrt{\varepsilon _1k_0^2-\left( k_1^{(\alpha )}\right) ^2}\nonumber \\ \text {with } k_1^{(\alpha )}&\approx \frac{\pi }{d_1} {\left\{ \begin{array}{ll} (2\alpha +1) &{} \text {(TE)}\\ 2\alpha &{} \text {(TM)} \end{array}\right. }. \end{aligned}$$

If the fields laterally concentrate in the metal domain, the phase $\varphi _1$ has a large, negative imaginary part $\Im [\varphi _1]\,{\ll }\,{-}1$, leading to

$$\begin{aligned} \lambda (q)\approx Z_2\sin [\varphi _2]-\imath Z_1\cos [\varphi _2]. \end{aligned}$$

Since $\Vert k_1\Vert \,{\gg }\,\Vert k_2\Vert$, and $\Vert \varepsilon _1\Vert \,{\ll }\,\Vert \varepsilon _2\Vert$, we obtain $\Vert Z_1\Vert \,{\gg }\,\Vert Z_2\Vert$ irrespective of polarization. The approximate sequence of roots for both polarizations is thus

5 $$\begin{aligned} q_2^{(\alpha )}&= \sqrt{\varepsilon _2k_0^2-\left( k_2^{(\alpha )}\right) ^2}\nonumber \\ \text {with } k_2^{(\alpha )}&\approx \frac{\pi }{d_2}(2\alpha +1). \end{aligned}$$

**Fig. 2:**
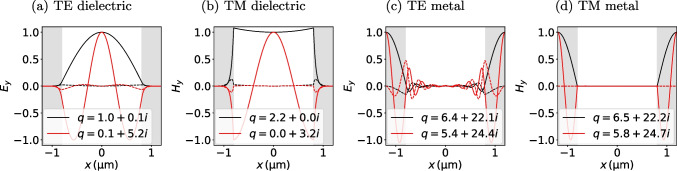
Floquet mode fields (1) for the single grating layer shown in Fig. 1c made of air and platinum with a lattice constant $d\,{=}\,{2.4}\,\upmu{\rm m}$ and a metal fill fraction of $\phi \,{=}\,0.33$ at vacuum wavelength $\lambda _0\,{=}\,{3}\,\upmu{\rm m}$. The unit cell is chosen such that $x\,{=}\,0$ lies in the center of the dielectric region with the metal domain shaded gray. The (a), (c) TE fields $E_y$ and the (b), (d) TM fields $H_y$ are normalized to 1 in the center of the (a), (b) dielectric and (c), (d) metal majority domain. Shown is the real part (solid line) and imaginary part (dashed line) of the fundamental (black) and the first order (red) field with the corresponding wave number *q* (in $\upmu{\rm m}$﻿)

**Fig. 3:**
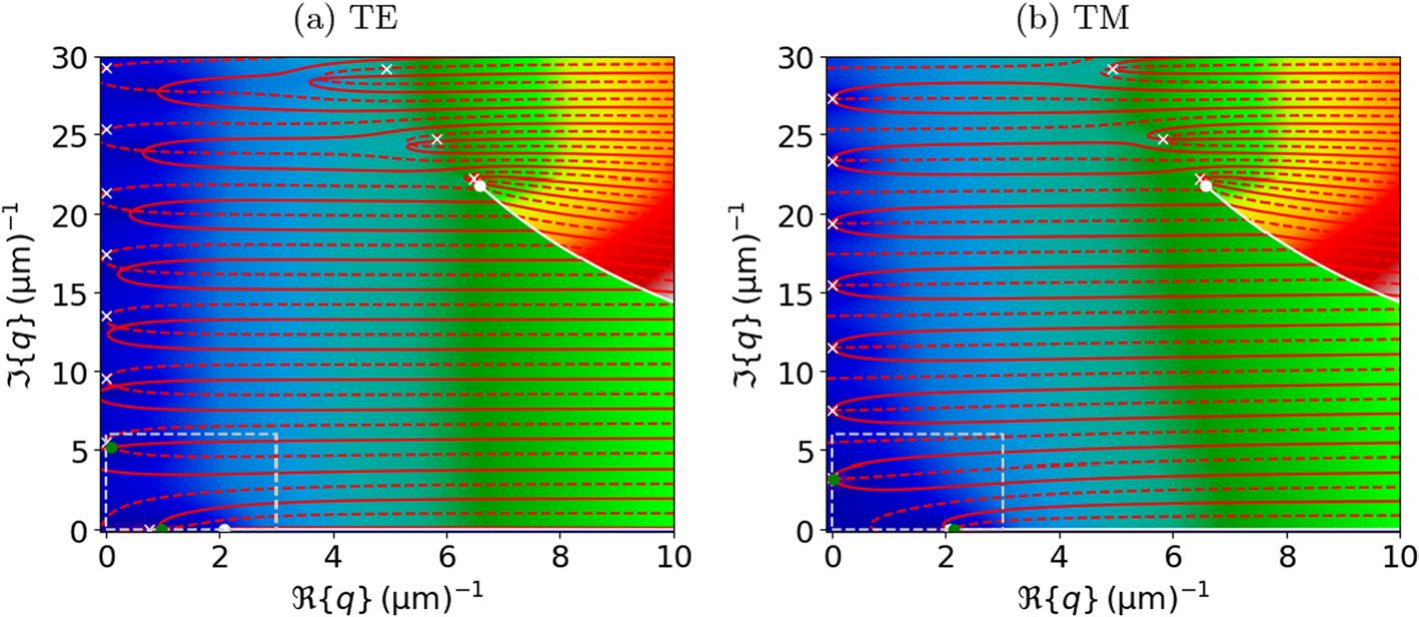
Visualization of $\lambda (q)$ in (3) for the same parameters as in Fig. 2. The heatmap shows the logarithmic absolute value from zero (dark blue) to large values (red). $\lambda$ inherits two branch points (white dots), with corresponding branch cuts (white lines) from the $k_{\eta }$ root functions. The approximate solutions (4) and (5) are indicated by white crosses, while the exact roots are where the $\Re \{\lambda \}\,{=}\,0$ (solid red line) and the $\Im \{\lambda \}\,{=}\,0$ (dashed red line) contours cross. Numerically, the first two air roots were found at the green points using a contour integration along the dashed line

In the remainder of this paper, we only consider the fundamental TE/TM air mode with $\eta \,{=}\,\alpha \,{=}\,0$, which is sufficient to qualitatively explain the observed scattering physics for small metal fill fractions^1^ and a lateral lattice constant below the first Wood anomaly ($a\,{<}\,\lambda$). The successful application of this crude approximation can be understood by considering the following two facts: First, higher-order dielectric modes have little intensity in the fundamental Bragg scattering order (the DC Fourier component in *x*). On the other hand, the metal modes exhibit a weak coupling to a vacuum plane wave due to the strong impedance mismatch. The exact roots of the fundamental mode can be found using a standard Newton procedure with (4) as an initial estimate. The fundamental and first-order modes of a platinum-air grating are shown in Fig. 2. Higher-order TE modes, where the Newton formalism does not converge with the analytical guess values, can be obtained using a global contour-integral method on a disk in the complex plane around the approximate higher-order root positions [51]. We illustrate the behavior of $\lambda (q)$, including the branch cuts, the position of the roots, and the contour integral method in Fig. 3.

### Chiral woodpile unit cell transfer matrix and complex band structure

We conveniently calculate the transfer matrix within one unit cell using the single layer transfer matrix ${\mathcal {T}}$ and the rotation matrix ${\mathcal {R}}$ between neighboring layers, as illustrated in Fig. 1d. The layer transfer matrix connects the parallel components of the fields $(E_x,E_y,H_x,H_y)$ at the top of the layer to those at its bottom in its native coordinate frame, shown in Fig. 1c, d.

We first introduce the impedance matrix *Z* that translates from the wave amplitudes of the Floquet states $\textbf{f}\,{:=}\,(f_\mathrm{{TE}}^{(+)},f_\mathrm{{TM}}^{(+)},f_\mathrm{{TE}}^{(-)},f_\mathrm{{TM}}^{(-)})$, containing the TE/TM amplitudes of the downward ($+$, with group velocity in positive *z* direction) and upward (–) waves $f_\mathrm{{TE/TM}}^{(\pm )}$, to the parallel field components in the local coordinate frame.^2^ It is thus given by

$$\begin{aligned} Z = \begin{pmatrix} 0 &{} \frac{k_0}{q_\mathrm{{TM}}} &{} 0 &{} -\frac{k_0}{q_\mathrm{{TM}}} \\ 1 &{} 0 &{} 1 &{} 0 \\ -\frac{q_\mathrm{{TE}}}{k_0} &{} 0 &{} \frac{q_\mathrm{{TE}}}{k_0} &{} 0 \\ 0 &{} 1 &{} 0 &{} 1 \end{pmatrix}, \end{aligned}$$

with the $q_\mathrm{{TE}}$ and $q_\mathrm{{TM}}$ the respective wave number of the fundamental Floquet mode within the air region $q_1^{(0)}$, see (4). The corresponding propagation matrix for the Floquet states is

$$\begin{aligned} P\,{:=}\,\text {diag}(p_\mathrm{{TE}}^{(+)},p_\mathrm{{TM}}^{(+)},p_\mathrm{{TE}}^{(-)},p_\mathrm{{TM}}^{(-)}), \end{aligned}$$

with $p_\mathrm{{TE/TM}}^{(\pm )}\,{:=}\,\exp \{\pm \imath q_\mathrm{{TE/TM} }h\}$. Using the impedance and the propagation matrix defined above, we thus obtain for the layer transfer matrix

6 $$\begin{aligned} {\mathcal {T}}\,{=}\,Z.P.Z^{-1}. \end{aligned}$$

The rotation matrix between neighboring layers is

7 $$\begin{aligned} {\mathcal {R}} = \mathbbm {1} \otimes \begin{pmatrix} \cos \theta &{} -\sin \theta \\ \sin \theta &{} \cos \theta \end{pmatrix} \end{aligned}$$

with the $2\times 2$ identity matrix $\mathbbm {1}$ and the Kronecker product $\otimes$. The rotation angle is generally $\theta \,{=}\,2\pi /N$ ($N\,{\in }\,{\mathbb {N}}$), with $N\,{=}\,3$ for the hexagonal woodpile with full crystallographic symmetry discussed here. The corresponding unit cell transfer matrix is

8 $$\begin{aligned} {\mathcal {T}}_\mathrm{{UC}} = ({\mathcal {T}}.{\mathcal {R}})^N. \end{aligned}$$

The eigen decomposition

9 $$\begin{aligned} {\mathcal {T}}_\mathrm{{UC}}\,{=}\,V.\Lambda .V^{-1}, \end{aligned}$$

with a diagonal matrix $\Lambda \,{=}\,\text {diag}(\lambda _\beta )$, trivially yields the complex Floquet band structure with the complex wave number

10 $$\begin{aligned} \kappa _\beta = \frac{1}{\imath c} \ln (\lambda _\beta ). \end{aligned}$$

The corresponding eigenfield to the Floquet wave number $\kappa _\beta$ is obtained through re-substituting the field components, given by the $\beta$-th column of $Z^{-1}.V$, into (1).

### Chiral woodpile scattering matrix

We here connect the Floquet states belonging to the complex band structure (10) to the scattering parameters reflectivity, transmissivity, and absorbance. We assume a slab of chiral woodpile on a substrate with isotropic (but generally frequency dependent) refractive index $n_\mathrm{{s}}$, and a circularly polarized plane-wave excitation at normal incidence from the top (assumed to be vacuum).

We first introduce the impedance matrix $Z_0(n)$ that translates from the circularly polarized plane-wave amplitudes $(f_{++},f_{-+},f_{+-},f_{--})$ within a background with refractive index *n* to the fields at an the interface at $z\,{=}\,0$ in the native coordinate frame, such that

$$\begin{aligned} \textbf{E}&= \sum _{\sigma _1=\pm }\sum _{\sigma _2=\pm } f_{\sigma _1\sigma _2} \left( \textbf{e}_x+\sigma _1\imath \textbf{e}_y\right) e^{\imath \sigma _2 n k_0 z}\quad \text {and}\\ \textbf{H}&= \sum _{\sigma _1=\pm }\sum _{\sigma _2=\pm } f_{\sigma _1\sigma _2} \sigma _2 n \left( -\imath \sigma _1\textbf{e}_x+\imath \textbf{e}_y\right) e^{\imath \sigma _2 n k_0 z}. \end{aligned}$$

The impedance matrix is thus given by

11 $$\begin{aligned} Z_0(n) = \begin{pmatrix} 1 &{} 1 &{} 1 &{} 1 \\ \imath &{} -\imath &{} \imath &{} -\imath \\ -\imath n &{} \imath n &{} \imath n &{} -\imath n \\ n &{} n &{} - n &{} - n \end{pmatrix}. \end{aligned}$$

Naively, the transfer matrix through a finite slab of *M* unit cells of chiral woodpile is thus given by

12 $$\begin{aligned} {\mathcal {T}}_\mathrm{{slab}} = Z_0^{-1}(n_s).{\mathcal {T}}_\mathrm{{UC}}^M.Z_0(1). \end{aligned}$$

Using (8) for the unit cell transfer matrix, this expression cannot compute scattering at a semi-infinite slab without substrate, becomes highly inefficient for thick slabs, and most importantly does not reveal the relation to the complex band structure. Using the eigen decomposition (9), however, the slab transfer matrix becomes

13 $$\begin{aligned} {\mathcal {T}}_\mathrm{{slab}} = Z_0^{-1}(n_s).V.\Lambda ^M.V^{-1}.Z_0(1), \end{aligned}$$

and for a semi-infinite slab

$$\begin{aligned} {\mathcal {T}}_\mathrm{{inf}} = V^{-1}.Z_0(1). \end{aligned}$$

To extract physical meaning, we need to translate these transfer matrices into the corresponding scattering matrices. For this, we first sort the 4 Floquet solutions in (9) with the permutation $\Pi$, such that the two waves propagating energy in positive *z* direction are stored first, and $\Lambda _\Pi \,{:=}\,\Pi .\Lambda .\Pi ^\intercal$ and $V_\Pi \,{:=}\,V.\Pi ^\intercal$.^3^ We re-express

14 $$\begin{aligned} {\mathcal {T}}_\mathrm{{inf}} = V_\Pi ^{-1}.Z_0(1), \end{aligned}$$

in the sorted basis and can now subdivide all transfer matrices into $2\,{\times }\,2$ sub-blocks that connect downward ($+$) and upward (−) moving amplitudes, respectively:

15 $$\begin{aligned} \begin{pmatrix} \textbf{f}_+^{(2)}\\ \textbf{f}_-^{(2)} \end{pmatrix} = \begin{pmatrix} {\mathcal {T}}_{++} &{} {\mathcal {T}}_{+-} \\ {\mathcal {T}}_{-+} &{} {\mathcal {T}}_{--} \end{pmatrix}\, \begin{pmatrix} \textbf{f}_+^{(1)}\\ \textbf{f}_-^{(1)} \end{pmatrix}. \end{aligned}$$

For the scattering matrix we use the most efficient convention [52] to relate the incoming to the outgoing amplitudes:

16 $$\begin{aligned} \begin{pmatrix} \textbf{f}_-^{(1)}\\ \textbf{f}_+^{(2)} \end{pmatrix} = \begin{pmatrix} {\mathcal {S}}_{11} &{} {\mathcal {S}}_{12} \\ {\mathcal {S}}_{21} &{} {\mathcal {S}}_{22} \end{pmatrix}\, \begin{pmatrix} P_1\,\textbf{f}_+^{(1)}\\ P_2\,\textbf{f}_-^{(2)} \end{pmatrix}. \end{aligned}$$

Note that we have included the $2\,{\times }\,2$ phase matrices that transport the incoming amplitudes from the other end of a finite slab to the interface in question for numerical stability [53, 54]. Since we are interested in intensities only, these are the identity matrices in the sub- and superstrate, and the respective sub-blocks of $\Lambda _\Pi ^M$ within the chiral woodpile. We can thus express the scattering matrix in terms of the transfer matrix as

17a $$\begin{aligned} {\mathcal {S}}_{11}&= -{\mathcal {T}}_{--}^{-1}.{\mathcal {T}}_{-+}.P_1 \end{aligned}$$

17b $$\begin{aligned} {\mathcal {S}}_{12}&= {\mathcal {T}}_{--}^{-1}.P_2 \end{aligned}$$

17c $$\begin{aligned} {\mathcal {S}}_{21}&= {\mathcal {T}}_{++}.P_1 + {\mathcal {T}}_{+-}.{\mathcal {S}}_{11} \end{aligned}$$

17d $$\begin{aligned} {\mathcal {S}}_{22}&= {\mathcal {T}}_{+-}.{\mathcal {S}}_{12}. \end{aligned}$$

For the semi-infinite slab, we substitute ${\mathcal {T}}_\mathrm{{inf}}$ and $P_1\,{=}\,\mathbbm {1}$ into (17a) to obtain the reflectivity matrix in the circular polarization basis $R\,{=}\,\Vert S_{11}\Vert ^2$.^4^ Energy conservation yields the absorptivity $A_\mathrm{{inf}}^{(\sigma )}\,{=}\,1{-}\sum _{\sigma '}R_{\sigma '\sigma }$ for left ($\sigma \,{=}\,{-}$) and right ($\sigma \,{=}\,{+}$) circularly polarized incoming light (from the point of view of the receiver).

**Table 1:** Investigated metals for the chiral plasmonic woodpile, including their permittivity data sources. For each metal, we calculated the maximum of the average CD over the spectral range $\Omega$ spanning from 75 to 100 THz, obtained for a semi-infinite PC of fixed layer pitch $d\,{=}\,{2.4}\, \upmu {\text {m}}$, and variable volume fill fraction of the metal $\phi$ and height of a single layer *h* (cf. Fig. 1). The average CD at the optimized position decreases for a finite slab of 15 unit cells on a substrate made of glass ($n_\mathrm{{s}}\,{=}\,1.5$), silicon ($n_\mathrm{{s}}\,{=}\,3.5$), or the metal in question (m)

Material	Source	$d_1 [\upmu {\text {m}}]$	$h [\upmu {\text {m}}]$	$\langle \textrm{CD}\rangle _\mathrm{{inf}}$	$\langle \textrm{CD}\rangle _{1.5}$	$\langle \textrm{CD}\rangle _{3.5}$	$\langle \textrm{CD}\rangle _\mathrm{{m}}$
Platinum (Pt)	[55]	1.94	0.68	0.84	0.58	0.73	0.71
Aluminum (Al)	[56]	1.98	0.69	0.91	0.39	0.63	0.31
Iron (Fe)	[56]	1.92	0.68	0.78	0.66	0.73	0.75
Tungsten (W)	[56]	1.98	0.68	0.89	0.46	0.68	0.49

For thick finite slabs, the transfer matrix ${\mathcal {T}}_\mathrm{{slab}}$ in (12) becomes numerically ill-conditioned. This problem is well-known [53], and even exists in the fundamental Bragg order here due to the possible strong evanescence of the fundamental Floquet modes (see Sect. 3). As a consequence, the application of (17) to the slab transfer matrix in (12) fails in practice. Instead, the scattering matrix ${\mathcal {S}}_\mathrm{{t}}$ at the top interface between the vacuum and the woodpile is well behaved and obtained by substituting ${\mathcal {T}}\,{=}\,V_\Pi ^{-1}.Z_0(1)$, $P_1\,{=}\,\mathbbm {1}$, and $P_2\,{=}\,\Lambda _{\Pi ,-}^{-M}$ into (17). Similarly, the scattering matrix ${\mathcal {S}}_\mathrm{{b}}$ between the woodpile and the substrate is obtained by substituting ${\mathcal {T}}\,{=}\,Z_0^{-1}(n_s).V_\Pi$, $P_1\,{=}\,\Lambda _{\Pi ,+}^M$, and $P_2\,{=}\,\mathbbm {1}$ into (17). The scattering matrix through an arbitrary finite slab of chiral woodpile is hence obtained in a numerically well-behaved way through the application of the Redheffer star product [54, 57]

18 $$\begin{aligned} {\mathcal {S}}_\mathrm{{slab}} = {\mathcal {S}}_\mathrm{{t}}\circledast {\mathcal {S}}_\mathrm{{b}}, \end{aligned}$$

defined for $2N\,{\times }\,2N$ matrices $C\,{=}\,A\circledast B$ with $N\,{\times }\,N$ sub-blocks as

$$\begin{aligned} C_{11}&:= A_{11} + A_{12}\left( \mathbbm {1}-B_{11}A_{22}\right) ^{-1}B_{11}A_{21} \\ C_{12}&:= A_{12}\left( \mathbbm {1}-B_{11}A_{22}\right) ^{-1}B_{12} \\ C_{21}&:= B_{21}\left( \mathbbm {1}-A_{22}B_{11}\right) ^{-1}A_{21} \\ C_{22}&:= B_{22} + B_{21}\left( \mathbbm {1}-A_{22}B_{11}\right) ^{-1}A_{22}B_{12}. \end{aligned}$$

The finite slab reflectivity matrix is then $R\,{=}\,\Vert ({\mathcal {S}}_\mathrm{{slab}})_{11}\Vert ^2$, while the transmissivity matrix is $T\,{=}\,n_s \Vert ({\mathcal {S}}_\mathrm{{slab}})_{21}\Vert ^2$. As for the semi-infinite woodpile, energy conservation yields the absorptivity. If the substrate is lossless, we obtain $A_\mathrm{{slab}}^{(\sigma )}\,{=}\,1{-}\sum _{\sigma '}\left( R_{\sigma '\sigma }{+}T_{\sigma '\sigma }\right)$ for left ($\sigma \,{=}\,{-}$) and right ($\sigma \,{=}\,{+}$) circularly polarized incoming light. For a lossy substrate, we instead have $A_\mathrm{{slab}}^{(\sigma )}\,{=}\,1{-}\sum _{\sigma '}R_{\sigma '\sigma }$. We generally define the (spectral) circular dichroism, both for the slab and the semi-infinite chiral woodpile, as

19 $$\begin{aligned} \text {CD}(\omega ) = \sum _\sigma \sigma \,A^{(\sigma )}, \end{aligned}$$

and the spectrally averaged circular dichroism, averaged over the frequency range $\Omega$ (from approximately 75THz to 100THz) as

20 $$\begin{aligned} \langle \text {CD}\rangle = \frac{1}{\int _\Omega \textrm{d}\omega }\int _\Omega \textrm{d}\omega \,\text {CD}(\omega ). \end{aligned}$$

### full-wave simulations and materials

**Fig. 4:**
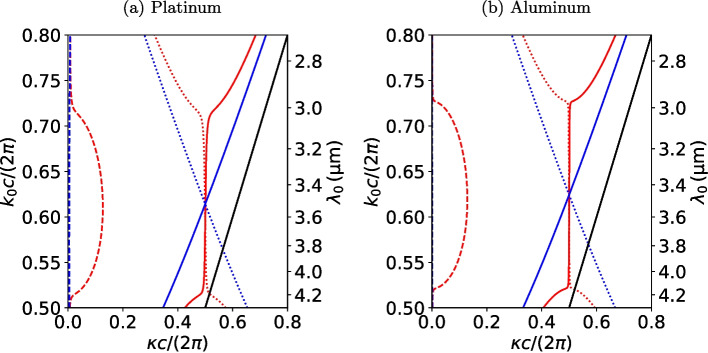
Complex band structures of the two fundamental Floquet modes in an extended Brillouin zone for a chiral woodpile made of (a) platinum and air, and (b) aluminum and air, with $d\,{=}\,{2.4}\, \upmu {\text {m}}$, metal fill fraction $\phi \,{=}\,0.2$, and $h\,{=}\,{0.72}\, \upmu {\text {m}}$ (Fig. 1). The solid lines show the real part of the wave number of the right (red) and left (blue) circular polarization-philic mode. The corresponding imaginary parts are shown as dashed lines (note that the dashed blue line is invisible for Al as $\Im \{\kappa \}\,{<}\,10^{-2}$ over the spectral range). The real parts of the modes propagating in ${-}z$ direction (with a negative imaginary part) are shown as dotted lines. The solid black line is the light line

The semi-analytical results have been compared to full-wave Maxwell simulations using COMSOL Multiphysics. The frequency domain simulations were performed on a lateral hexagonal unit cell, as shown in Fig. 1a, employing periodic boundary conditions and a tetrahedral finite element mesh with second order tetrahedral elements and a maximum edge length of *a*/10. We thus simulated a finite woodpile structure for optimized geometrical parameters and 15 unit cells slab thickness. An air domain and a substrate domain of 2*c* height were added above and below the woodpile, respectively. These vacuum and substrate domains were terminated by periodic ports, in which the system was excited through the vacuum port in the circularly polarized basis. The ${\mathcal {S}}$-parameters were extracted from the Rayleigh components of the fields at these ports.

The material parameters for the different metals were taken from the refractive index database [58]. We specifically investigated the plasmonic metals summarized in Table 1. As the substrate, we used fused silica glass with $n_\mathrm{{s}}\approx 1.5$, silicon with $n_\mathrm{{s}}\approx 3.5$, which were approximated as non-dispersive and lossless materials, and the specific metal in question with strongly dispersive refractive index.

## Results and discussion

### Complex band structure of the chiral plasmonic woodpile

To understand the guiding principle behind light propagation in the chiral plasmonic woodpile structures, we start analyzing the evanescent Floquet states calculated by the algorithm introduced in Sect. 2.2. For a broad range of geometrical parameters, which will be discussed in more detail in Sect. 3.2, we find a *polarization bandgap* in the frequency range of interest $\Omega$ between 3 and 4 $\upmu$m wavelength: On the one hand, the two fundamental Floquet modes either predominantly couple to left (LCP-philic) or right (RCP-philic) circular polarization. On the other hand, the LCP-philic mode exhibits a band structure with weak dispersion and a small imaginary part, while the RCP-philic mode gives rise to a large bandgap with a relatively large imaginary part and a real part close to the Brillouin zone boundary at $\kappa \,{\approx }\,\pi /c$, as shown in Fig. 4.

While the polarization bandgap resembles that of a chiral high index photonic crystal, there are two distinct differences: First, the PC bandgap found here is above the frequency $k_0\,{=}\,\pi /c$, while in a photonic crystal, it is below that frequency, simplifying top-down fabrication as the corresponding structures for a mid-IR target frequency will be bigger. Second, the RCP-philic mode is not exactly pinned to the Brillouin zone boundary at $\kappa \,{=}\,\pi /c$, corresponding to a non-vanishing energy propagation and thus the expected finite energy loss of the strongly evanescent mode within the structure. Similarly, the LCP-philic mode has a small, but non-vanishing imaginary part of $\kappa$, corresponding to a small evanescence or Beer–Lambert-like energy dissipation while propagating through the woodpile PC. As we will demonstrate in Sect. 3.3, the attenuation is underestimated by our approximate theory, as we do not consider the higher-order Floquet modes that concentrate their energy in the metal domain and thus give rise to an additional loss in the PC.

Regarding the circular polarization discrimination, the field polarization of the two Floquet states resides close to the north and south poles of the Poincaré sphere, respectively. We quantify the circular dichroism of the Floquet states with the circular dichroism index [12, 59]

21 $$\begin{aligned} {\mathcal {C}} = \frac{C_+ - C_-}{C_+ + C_-}, \end{aligned}$$

that considers the relative difference in coupling to the two circularly polarized plane waves and the Floquet field and ranges from 0 (no difference) to $\pm 1$ (maximum difference). The total in-coupling from the vacuum is similarly quantified through the coupling index

22 $$\begin{aligned} \beta = C_+ + C_-, \end{aligned}$$

that ranges from 0 (no in-coupling) to 1 (maximum in-coupling). The individual RCP and LCP couplings in these expressions are approximated by

$$\begin{aligned} C_{\pm } = \frac{1}{4\Vert V_\Pi ^{(i)}\Vert ^2}\left\| \begin{pmatrix} 1 \\ \mp \imath \\ \pm \imath \\ 1 \end{pmatrix}\cdot V_\Pi ^{(i)}\right\| ^2, \end{aligned}$$

where $V_\Pi ^{(i)}$ ($i\,{=}\,1,2$) is the *i*th sorted eigenvector of the unit cell transfer matrix, that is the *i*th column of $V_\Pi$, which contains the lateral electromagnetic fields of the corresponding Floquet state on the $C_{6_2}$ axis at the top of the unit cell. As this definition considers both the electric and magnetic fields, it takes the impedance match into account.

We find that the circular dichroism index for the platinum structure corresponding to the band structure in Fig. 4 at wavelengths $\lambda _0\,{=}\,(3,3.5,4)\,\upmu{{\text {m}}}$ is ${\mathcal {C}}\,{=}\,(-0.84,-0.83,-0.81)$ for the LCP-philic mode and ${\mathcal {C}}\,{=}\,(0.98,0.94,0.89)$ for the RCP-philic mode. In other words, incoming LCP light couples almost exclusively into the blue, weakly attenuated propagating Floquet state, while RCP light couples predominantly into the red, strongly evanescent state in Fig. 4a, justifying the color assignment. Further, the coupling index is $\beta \,{=}\,(0.99,0.99,0.98)$ into the LCP-philic mode, and $\beta \,{=}\,(0.67,0.52,0.52)$ into the RCP-philic mode for $\lambda _0\,{=}\,(3,3.5,4)\,\upmu{{\text {m}}}$. Even though the approximate theory is expected to overestimate the coupling, this suggests that almost all of the incoming LCP light is transmitted into the woodpile PC and absorbed through Beer attenuation within. On the other hand, a considerable amount of RCP light is expected to couple into the red, strongly evanescent Floquet mode. Since this mode, however, propagates almost no energy into the structure within the bandgap region, RCP light is mainly reflected with little absorption.

### Optimizing broadband circular dichroism

**Fig. 5:**
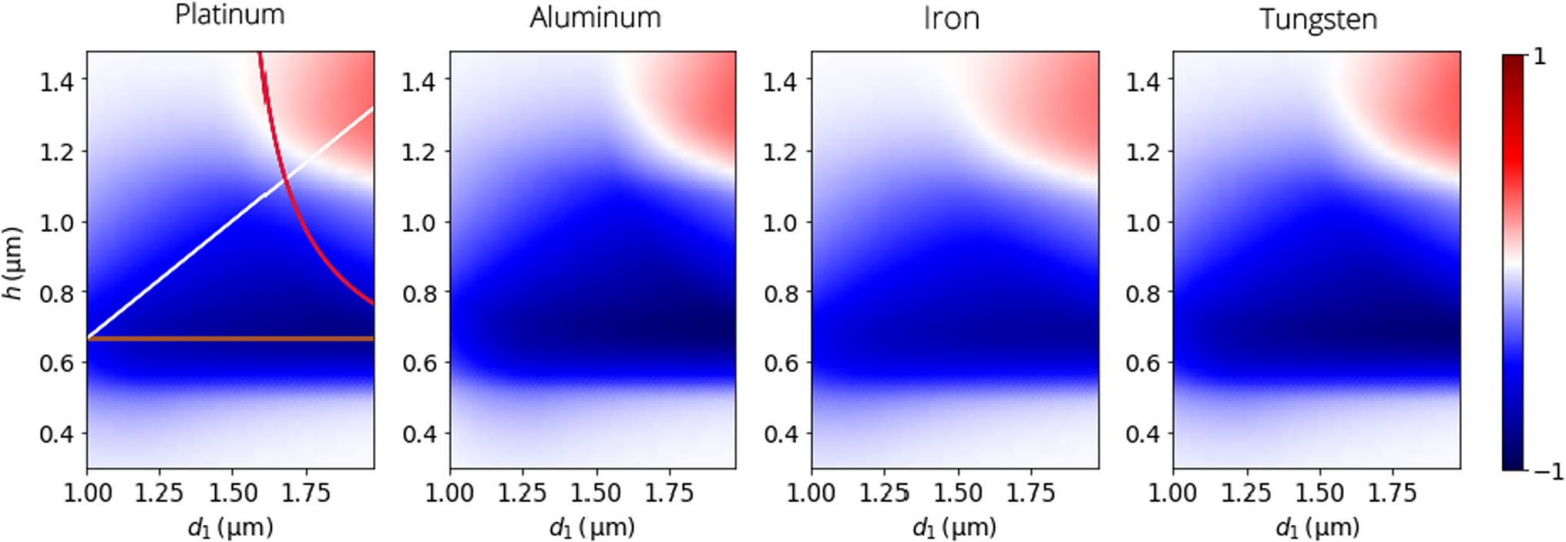
$\langle \textrm{CD}\rangle _\mathrm{{inf}}$, as defined in (20), for four different semi-infinite metal-air chiral woodpile PCs with $d\,{=}\,{2.4}\, \upmu {\text {m}}$ as a function of dielectric region width $d_1$ and layer height *h*, see Fig. 1 c). The three lines indicate the theoretical limits of the high CD region based on the simplified Bouligand model discussed in the main text

While the qualitative chiro-optical behavior of the chiral woodpile PC is well understood within the band structure picture discussed in Sect. 3.1, we here perform a more quantitative analysis calculating the scattering matrix and the circular dichroism as outlined in Sect. 2.3.

Next to revealing the main physical scattering mechanism, the semi-analytical Floquet mode algorithm comes at a very low numerical cost. The scattering parameters for one wavelength are calculated within 2 ms (in a non-optimized Python implementation), compared to 5 min for the full-wave simulations (see Sect. 2.4 for details). This efficiency enables a brute-force scan over geometrical parameters for different materials to extract the spectrally averaged circular dichroism $\langle \textrm{CD}\rangle$, defined in (20). We find, that a strong CD can be generally observed for lattice constants below, but on the order of 3 $\upmu{{\text {m}}}$, and have therefore fixed the single layer pitch to $d\,{=}\,{2.4}\,\upmu {{\text {m}}}$ ($a\,{\approx }\,{2.77}\,\upmu {{\text {m}}}$) for the remainder of this manuscript.

For the four metals listed in Table 1, we have calculated the average CD of the semi-infinite metal-air PC with varying thickness of the in-plane air region $d_1\,{\in }\,[1,2]\,\upmu {{\text {m}}}$ and the layer height $h\,{\in }\,[0.3,1.5]\,\upmu {{\text {m}}}$. Note that the metal fill fraction and the chiral pitch are hence varied according to $\phi \,{=}\,1{-}d_1/d$ and $c\,{=}\,3h$. The results illustrated in Fig. 5 reveal a broad region within the parameter space, where a substantial CD can be observed, largely independent of the chosen metal. This region between 600 and 800 nm layer heights, where the spectrally averaged CD is above 0.5, spans almost all $d_1$, with a slight increase towards larger thicknesses. While aluminum and tungsten yield the strongest CD for the semi-infinite slab (cf. Table 1), their more strongly perfect plasmonic nature ($\Im \{n\}\,{\gg }\,\Re \{n\}$) gives rise to very little field penetration into the metal region. This in turn makes them less efficient in more realistic finite slabs, since the attenuation of the LCP-philic mode (Sect. 3.1) and thus LCP absorption is much weaker. As discussed in Sect. 3.3, attenuation is, however, underestimated by our theoretical approximation. The chiral PC is expected to yield a substantial spectrally averaged CD, (20), of 70% for all metals, insensitive to fabrication imperfections. A specific metal alongside geometrical parameters within a broad region may hence be chosen depending on the limitations of a specific fabrication routine.

In summary, the CD mainly depends on $d_1$ and *h* (and not explicitly on *a*) and is strongest within a specific region in Fig. 5, which is independent of the metal in question. To better understand this behavior, we approximate the chiral woodpile even further by replacing it by a plasmonic version of a semi-continuous Bouligand structure, as found in liquid crystals and biological systems [60]. Since we expect the electromagnetic field to rotate with the smaller 60$^\circ$ rotation of the $C_6$ screw rotation, and not the 120$^\circ$ of the $C_3$ rotation used in Sect. 2.2 and illustrated in Fig. 1, we build the Bouligand structure such that it rotates in the opposite direction and has a pitch of 2*c*. The anisotropic lateral (*x*-*y*) permittivity matrix depending on $\varepsilon _\mathrm{{TE/TM}}\,{:=}\,q_\mathrm{{TE/TM}}/k_0$ at height *z* is in the Bouligand picture expressed by:

23 $$\begin{aligned} \varepsilon (z)&= R(z).\textrm{diag}(\varepsilon _\mathrm{{TE}},\varepsilon _\mathrm{{TM}}).R^{-1}(z), \, \text { with},\nonumber \\ R(z)&:= \begin{pmatrix} \cos (Gz) &{} \sin (Gz) \\ -\sin (Gz) &{} \cos (Gz) \end{pmatrix} \nonumber \\ \text {and }G&:=\pi /c. \end{aligned}$$

The monochromatic Maxwell equations can be solved with the ansatz

24 $$\begin{aligned} R(z).\textbf{E}_0\,e^{\imath \kappa z}, \end{aligned}$$

for the lateral electric field (with $E_z\,{=}\,0$). With the approximate solutions for the Floquet wave numbers (4), this procedure yields the following 2D quadratic eigenproblem in $\kappa$:

25 $$\begin{aligned} \begin{pmatrix} \kappa ^2{+}G^2{+}G_1^2{-}k_0^2 &{} {-}2\imath G\kappa \\ 2\imath G\kappa &{} \kappa ^2{+}G^2{-}k_0^2 \end{pmatrix}.\textbf{E}_0 = 0 \end{aligned}$$

with $G_1\,{:=}\,\pi /d_1$. One can immediately see, that this approximate Bouligand equation only depends on the chiral pitch *c* (or layer height *h*) and thickness of the air region $d_1$.

Let us first discuss the solution to (25) for $k_0 \,{=}\,G$, for which the characteristic equation yields eigenpairs $(\kappa ,\textbf{E}_0)$ for the downward propagating waves

$$\begin{aligned}&\left( \kappa _\mathrm{{R}} = 0 ,\textbf{E}_{0\textrm{R}} = \textbf{e}_y\right) \\ \text {and}\quad&\left( \kappa _\mathrm{{L}} = \sqrt{4G^2-G_1^2},\textbf{E}_{0\textrm{L}} =\textbf{e}_x - \imath \frac{2G}{\kappa _\mathrm{{L}}}\textbf{e}_y\right) \text { .} \end{aligned}$$

The R branch corresponds to the lower end of the bandgap in Fig. 4 with $\kappa _\mathrm{{R}}\,{\approx }\,0$.^5^ The mode is, however, linearly polarized in *y* direction. On the other hand, the LCP-philic solution is indeed left elliptically polarized, if $\kappa _\mathrm{{L}}$ is real, that is if $h\,{<}\,\frac{2}{3}d_1$. At the upper end of the bandgap at $k_0\,{=}\,\sqrt{G^2+G_1^2}$ we obtain a similar solution

$$\begin{aligned}&\left( \kappa _\mathrm{{R}} = 0 , \textbf{E}_{0\textrm{R}}=\textbf{e}_x\right) \\ \text {and}\quad&\left( \kappa _\mathrm{{L}} = \sqrt{4G^2+G_1^2}, \textbf{E}_{0\textrm{L}} =\textbf{e}_x - \imath \frac{\kappa _\mathrm{{L}}}{2G}\textbf{e}_y\right) \text { .} \end{aligned}$$

The R branch is now linearly polarized in *x* direction. While the L branch is always left elliptically polarized, it approaches circular polarization if $h\,{\ll }\,\frac{2}{3}d_1$. These two branches are connected by the solution within the center of the gap at $k_0\,{=}\,\sqrt{G^2+G_1^2/2}$:

$$\left( {\kappa _{ \pm } = \sqrt {2G^{2} \pm \sqrt {4G^{4} + \frac{{G_{1}^{4} }}{4}} } ,\,\,{\mathbf{E}}_{{0 \pm }} = {\mathbf{e}}_{x} - \imath \frac{{2G\kappa _{ \pm } }}{{\kappa _{ \pm }^{2} - G_{1}^{2} /2}}{\mathbf{e}}_{y} } \right).$$

Evidently, $\kappa _-$ is purely imaginary, as expected in the center of the bandgap. It connects the $\kappa _\mathrm{{R}}$ solutions and is linearly polarized with polarization direction between *x* and *y*. On the other hand, $\kappa _+$ belongs to the $\kappa _\mathrm{{L}}$ branch and is left elliptically polarized, quickly approaching circular polarization if $h\,{\ll }\,\frac{2}{3}d_1$.

The Bouligand model thus identifies three main contributors that limit the region of high average spectral CD. First, the $\kappa _\mathrm{{R}}$ bandgap needs to reside within the spectral range of interest. To keep the lower band edge outside the spectral region of interest, we require $G\,{<}\,2\pi /\lambda _\mathrm{{l}}$, with $\lambda _\mathrm{{l}}\,{=}\,{4}\, \upmu {\text {m}}$ in our case. This yields $h\,{>}\,\lambda _\mathrm{{l}}/6\,{\approx }\,{0.67}\, \upmu {\text {m}}$, shown as brown line in Fig. 5, which explains the horizontal border at the bottom of the high CD domain. Similarly, keeping the upper bandgap edge outside the spectral region of interest requires

$$\begin{aligned} h < \frac{d_1}{3\sqrt{4d_1^2/\lambda _0^2-1}}, \end{aligned}$$

(where the radicand is positive), shown as a red line in Fig. 5. Above this line, the red region in the heatmap indicates a sign change in the average CD. This change is caused by a dichroic color switch [61], meaning that there is an additional bandgap of opposite optical chirality at higher frequencies, which also exists in chiral woodpile photonic crystals [38] and moves into the spectral region of interest.

Finally, the polarization of the $\kappa _\mathrm{{L}}$ mode needs to resemble LCP polarization, i.e. it needs to be as close as possible to the pole of the Poincaré sphere, to yield a strong CD in the bandgap. This implies that we need to be as far as possible under the white $h\,{=}\,\frac{2}{3}d_1$ line in Fig. 5, explaining the less sharp positively sloped upper termination of the high CD region. While the Bouligand model can thus explain the general shape of the high CD region, it predicts the red bandgap mode to be linearly polarized in contrast to the hexagonal chiral woodpile bandgap modes, which are clearly right circularly polarized as demonstrated by the CD index in Sec. 3.1.

### Comparison to full-wave simulations

**Fig. 6:**
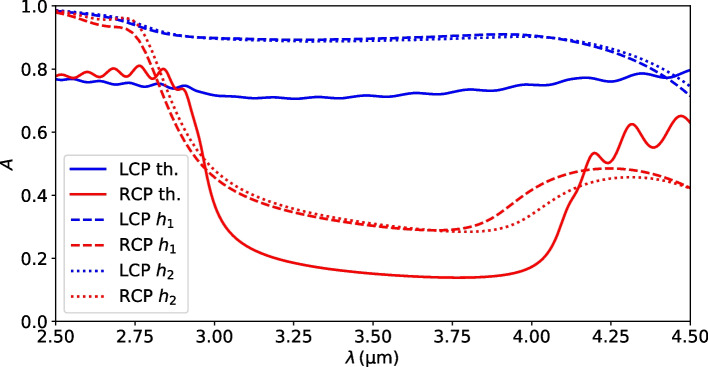
Theoretically predicted (solid lines) and simulated (dashed lines) absorption spectra under LCP (blue) and RCP (red) illumination for an iron-air chiral woodpile PC on a glass substrate ($n_\mathrm{{s}}\,{=}\,1.5$) with $d\,{=}\,{2.4}\, \upmu {\text {m}}$, $\varphi \,{=}\,0.2$, and $h_1\,{=}\,{0.7}\, \upmu {\text {m}}$. The right band edge red-shifts in the simulated absorption spectra if the layer height is slightly increased to $h_2\,{=}\,{0.72}\, \upmu {\text {m}}$ (dotted lines)

Considering the crude approximations made, the theory predicts the full spectral CD of all metals well. To demonstrate this, we have calculated the RCP and LCP absorption through full-wave simulations as described in Sec. 2.4. The results for an iron-air woodpile PC on a glass substrate with $d\,{=}\,{2.4}\, \upmu {\text {m}}$, $\varphi \,{=}\,0.2$, and $h_1\,{=}\,{0.7}\, \upmu {\text {m}}$, close to the optimal parameters of the semi-infinite slab, are shown in Fig. 6. Clearly, the position of the bandgap is predicted well, although the full-wave results seem to be slightly blue-shifted compared to the theory. Generally, the theory underestimates the absorption for both polarizations within the bandgap region. This behavior is expected, as we do not consider the single layer higher order fields that reside mainly in the metal domain. These lead to stronger absorption both in the evanescent Floquet mode (red spectrum), and the propagating mode (blue spectrum). An additional indication that absorption and hence attenuation in the propagating Floquet mode is increased in the simulations is the absence of the Fabry–Pérot interference pattern that is clearly visible in the theoretical spectrum. At high wavelengths above ${4}\, \upmu {\text {m}}$ on the other hand, where iron begins to act more and more like a perfect electrical conductor, the inclusion of the higher order metallic modes is expected to lead to an underestimated impedance mismatch between the incoming vacuum field and the Floquet states within. The theory, therefore, underestimates the reflection and overestimates the absorption for both polarizations alike.

While the theory cannot accurately predict the spectra, we have thus demonstrated that all qualitative physical predictions made in the preceding chapters are accurate and can be used to tailor a chiral woodpile PC to a specific application and fabrication procedure. These general principles can also help to further fine-tune the geometry to improve the non-approximated CD. For example, the position of the bandgap in Fig. 6 seems to be too far to the left. This suggests increasing the layer height *h* slightly, which is predicted to red-shift the right band edge towards 4 $\upmu$m, while only weakly affecting the left edge at 3 $\upmu$m. We demonstrate this effect by simulating the same structure with an increased $h_2\,{=}\,{0.72}\, \upmu {\text {m}}$, shown as dotted line in Fig. 6.

## Conclusion

In conclusion, we have identified the underlying physical principles of broadband circular dichroism in chiral plasmonic woodpile structures employing an approximate evanescent Floquet mode picture. Focusing on the transparency window of the atmosphere between 3 and 4$\upmu$m wavelength in the mid-infrared frequency region, we have found a broad circular polarization bandgap that exists within a large region of geometrical parameters and for a number of different metals used. Employing a semi-continuous Bouligand model, we extracted general design principles that predict the approximate size and shape of the region in the geometrical parameter space, where large broadband circular dichroism is expected.

On the one hand, our findings demonstrate that evanescent Floquet modes and the associated complex bandstructure form an invaluable tool to understand scattering at and wave propagation within slab-like plasmonic crystals, which combine interference-dominated physics known from photonic crystals and material-dispersion induced effects known from classical metamaterials. On the other hand, we provide a pathway to design a broadband, highly efficient circularly polarized thermal source in the mid-infrared region by application of Kirchhoff’s law. A large broadband CD can be engineered within a predictable, massive region in the geometrical parameter space. This region encompasses a variable aspect ratio of the metal bars, which ranges from a ratio of approximately 1 : 2 to 2 : 1. The typical lattice constant is smaller, but comparable to the wavelength of the light, approximately twice as big as in high-index dielectric structures, making top-down fabrication more feasible for the envisioned mid-infrared window between 3 and 4$\upmu$m wavelength.

Indeed, a number of fabrication routines have been reported to yield woodpile structures for geometrical parameters within the predicted high-CD region, ranging from layer-by-layer manufacturing [62, 63] to two-photon lithography techniques [64]. Recent advances in two-photon lithography make it possible to directly produce metallic structures [65], while all other methods can produce an inverse mold on a conducting substrate (for example indium tin oxide). In both cases, an electro-deposition routine [66] can replicate the woodpile PC in a metal of choice. Standard direct laser writing on the other hand typically produces a polymeric woodpile structure, for which an electroless plating routine can be employed [67, 68].

## Footnotes

1 The model is expected to produce good results if the thickness of the metal domain is above the effective penetration depth of the fields on the order of 100 nm as seen in Fig. 2, but such that $\phi \,{\ll }\,1$.

2 Note that this impedance matrix formally resembles that of a homogenized view, but the layer propagation constants and fields are exact so that the approximation lies in cutting the Fourier series in its fundamental order when matching the fields between neighboring layers, not within.

3 Since energy is lost within the chiral woodpile, this simply requires sorting by $\Vert \lambda _\beta \Vert$ in ascending order.

4 This simple expression is a consequence of all waves in the circularly polarized basis to be normalized such that their energy flow in propagation direction is twice the background index times the squared absolute value of the amplitude.

5 Note that the boundary of the Brillouin zone is back-reflected to the $\Gamma$ point at the center in the considered non-trivial unit cell with pitch 2*c*.
